# 

**Published:** 1910-12-17

**Authors:** 


Telegraphic Address: Ospedale, London.

THE MODERN MEDICAL AND INSTITUTIONAL JOURNAL.

Telephone Number: 2734 Gerrard.

NEW SERIES. No. 201, Vol. VIII.

No. 1273, Vol. XLIX.

SATURDAY, DEC. 17TH, 1910.

PRICE THREEPENCE

